# Evaluation of walking activity and gait to identify physical and mental fatigue in neurodegenerative and immune disorders: preliminary insights from the IDEA-FAST feasibility study

**DOI:** 10.1186/s12984-024-01390-1

**Published:** 2024-06-05

**Authors:** Chloe Hinchliffe, Rana Zia Ur Rehman, Clemence Pinaud, Diogo Branco, Dan Jackson, Teemu Ahmaniemi, Tiago Guerreiro, Meenakshi Chatterjee, Nikolay V. Manyakov, Ioannis Pandis, Kristen Davies, Victoria Macrae, Svenja Aufenberg, Emma Paulides, Hanna Hildesheim, Jennifer Kudelka, Kirsten Emmert, Geert Van Gassen, Lynn Rochester, C. Janneke van der Woude, Ralf Reilmann, Walter Maetzler, Wan-Fai Ng, Silvia Del Din, the IDEA-FAST Consortium

**Affiliations:** 1 Translational and Clinical Research Institute, Faculty of Medical Sciences, Newcastle University, The Catalyst, 3 Science Square, Room 3.27, Newcastle Upon Tyne, NE4 5TG UK; 2 Janssen Research & Development, High Wycombe, UK; 3 Let It Care, Rennes, France; 4 LASIGE, Faculdade de Ciências, Universidade de Lisboa, Lisbon, Portugal; 5 Open Lab, School of Computing, Newcastle University, Newcastle Upon Tyne, UK; 6 VTT, Visiokatu 4, 33720 Tampere, Finland; 7 Janssen Research & Development, Cambridge, USA; 8 Janssen Research & Development, Beerse, Belgium; 9 Pfizer, Thessaloniki, Central Macedonia Greece; 10 NIHR Newcastle Clinical Research Facility, Newcastle Upon Tyne, UK; 11 National Institute for Health and Care Research (NIHR) Newcastle Biomedical Research Centre (BRC), Newcastle University and The Newcastle Upon Tyne Hospitals NHS Foundation Trust, Newcastle Upon Tyne, UK; 12 The Newcastle Upon Tyne Hospitals NHS Foundation Trust, Newcastle Upon Tyne, UK; 13 George-Huntington-Institute, Muenster, Germany; 14 Department of Gastroenterology and Hepatology, Erasmus University Medical Center, Molewaterplein 40, 3015 GD Rotterdam, The Netherlands; 15 Department of Neurology, University Medical Center Schleswig-Holstein Campus, Kiel, Germany; 16 Medical Affairs, Takeda, Brussels, Belgium

**Keywords:** Real-world gait, Machine learning, Wearable devices, Walking, Fatigue, Digital health

## Abstract

**Background:**

Many individuals with neurodegenerative (NDD) and immune-mediated inflammatory disorders (IMID) experience debilitating fatigue. Currently, assessments of fatigue rely on patient reported outcomes (PROs), which are subjective and prone to recall biases. Wearable devices, however, provide objective and reliable estimates of gait, an essential component of health, and may present objective evidence of fatigue. This study explored the relationships between gait characteristics derived from an inertial measurement unit (IMU) and patient-reported fatigue in the IDEA-FAST feasibility study.

**Methods:**

Participants with IMIDs and NDDs (Parkinson's disease (PD), Huntington's disease (HD), rheumatoid arthritis (RA), systemic lupus erythematosus (SLE), primary Sjogren’s syndrome (PSS), and inflammatory bowel disease (IBD)) wore a lower-back IMU continuously for up to 10 days at home. Concurrently, participants completed PROs (physical fatigue (PF) and mental fatigue (MF)) up to four times a day. Macro (volume, variability, pattern, and acceleration vector magnitude) and micro (pace, rhythm, variability, asymmetry, and postural control) gait characteristics were extracted from the accelerometer data. The associations of these measures with the PROs were evaluated using a generalised linear mixed-effects model (GLMM) and binary classification with machine learning.

**Results:**

Data were recorded from 72 participants: PD = 13, HD = 9, RA = 12, SLE = 9, PSS = 14, IBD = 15. For the GLMM, the variability of the non-walking bouts length (in seconds) with PF returned the highest conditional R2, 0.165, and with MF the highest marginal R2, 0.0018. For the machine learning classifiers, the highest accuracy of the current analysis was returned by the micro gait characteristics with an intrasubject cross validation method and MF as 56.90% (precision = 43.9%, recall = 51.4%). Overall, the acceleration vector magnitude, bout length variation, postural control, and gait rhythm were the most interesting characteristics for future analysis.

**Conclusions:**

Counterintuitively, the outcomes indicate that there is a weak relationship between typical gait measures and abnormal fatigue. However, factors such as the COVID-19 pandemic may have impacted gait behaviours. Therefore, further investigations with a larger cohort are required to fully understand the relationship between gait and abnormal fatigue.

**Supplementary Information:**

The online version contains supplementary material available at 10.1186/s12984-024-01390-1.

## Background

Many people with neurodegenerative disorders (NDD) and immune-mediated inflammatory diseases (IMID) experience abnormal fatigue. For instance, abnormal fatigue has been reported in over 85% of those with systemic lupus erythematosus (SLE) [1, 2], 33% to 58% of those with Parkinson’s disease (PD) [3], 67% of people with primary Sjogren’s syndrome (PSS) [4], and over 41% of patients with rheumatoid arthritis (RA) showed clinically important levels of fatigue [5]. These symptoms can be debilitating for those who experience them and are key contributors to poor quality of life. Accordingly, a key goal of the IDEA-FAST consortium [6] is to explore and identify digital endpoints that provide reliable, objective, and sensitive evaluations of abnormal fatigue, which in turn will facilitate therapeutic development to alleviate these symptoms.

A common method for assessing fatigue is patient reported outcomes (PRO)s, where the patients answer questionnaires and diaries designed to record how the patient is feeling. This approach gives a snapshot view of fatigue, either during a one-off visit at the clinic or with regular at-home questionnaires. PROs, however, are subjective, susceptible to recall bias [7], and the measurement of granular changes over time requires high patient burden through repeated assessments. Furthermore, studies have shown that individuals who are sleep-deprived or are out of their circadian phase are prone to underestimating their fatigue-related impairments [8–11]. These issues are therefore worsened in cases where neurological functionality or sleep quality is impacted by an illness. Wearable devices may circumvent the pitfalls of PRO-based assessments since they could objectively and continuously monitor physiological changes related to physical and mental fatigue. Inertial measurement units (IMUs)—e.g., wearable devices comprising triaxial accelerometers and gyroscopes—are becoming an increasingly popular option for continuous remote monitoring, due to their affordability and ease of use in users’ natural environment. These recordings can be wirelessly transmitted to a device, such as a smartphone, where the signals can be processed, assessed, and reported to the user or clinicians.

Mobility (e.g., walking, gait) is considered as the 6th vital sign and represents an essential component of health and quality of life, being key to physical, mental, and social well-being [12]. Loss in mobility has been associated with morbidity, falls, dementia, cognitive decline, hospitalisations, mortality, and symptoms of chronic disorders [13–16]. As such, the current study will explore the relationship between gait characteristics and abnormal fatigue in people with NDD and IMID.

The current knowledge in the relationships between walking and abnormal fatigue in NDDs and IMIDs is very limited. Many studies exploring fatigue focus on muscle or exercise-induced fatigue in healthy participants with parameters such as accelerometer spectra [17], measures of acceleration, jerk, and posture [18–21], and temporal measures of gait (“micro” gait, e.g., gait speed, step time) [22, 23]. Most notably, the majority of existing literature has been conducted within in laboratory-based environment, where the participants are monitored whilst doing an instructed task. This includes the six-minute walking distance test (6-MWDT) to assess the impacts of fatigue on gait (exercise) capacity [24–28]; gait speed i.e., the ten-meter walking test (10-MWT) [24, 29–32]; an electronic walkway [33, 34]; and a predefined path for unaided walking [35]. We found three studies that explored fatigue in NDD or IMID specifically with free-living gait assessment. The first analysed physical activity over a seven-day period from a hip-worn tri-axial accelerometer in 123 participants with SLE [36]. Light and moderate/vigorous activity and moderate/vigorous activity periods > 10 min were identified from the accelerometer’s vector magnitude and compared to the participants’ Fatigue Severity Score (FSS). The second study investigated the impact of physical activity on non-motor symptoms with the Movement Disorder Society-Unified Parkinson’s Disease Rating Scale (MDS-UPDRS) in 45 PD participants with a hip-worn accelerometer for at least three days to record daily step count and assess sedentary behaviour, light physical activity, and moderate-to-vigorous activity [37]. In the third, participants with multiple sclerosis (MS)—134 fatigued and 76 non-fatigued—wore an accelerometer above the dominant hip for seven days to record light and moderate-to-vigorous activity counts, whilst reporting on their fatigue severity with FSS [38].

In many cases, the exploration of gait measures is limited to characteristics such as gait speed [31, 39], and ambulatory activity (step count) [30]. Other studies were more focussed on gait, but only explored different activity levels based on step count [37], vector magnitude [36], and activity counts [38]. Another explored 6-MWT, dynamic activity, number of postural transitions, and walking bouts longer than ten seconds [28]. Three studies included a more comprehensive analysis of macro (reflecting activity) and micro (reflecting discrete gait outcomes) gait characteristics within a laboratory: one study assessed the associations of Multidimensional Fatigue Inventory (MFI) with measures of pace, rhythm, variability, asymmetry, and postural control [33]; one analysed the cycle time, stride length, swing time, and double support time and their variability (coefficient of variation (CoV)) [34]; and one compared gait duration (10-MWT), gait speed (m/s), cadence (steps/min), and stride length (m) to the Parkinson FSS [32]. Furthermore, the participants explored by the studies in the literature typically include only healthy subjects [33] or only one disease cohort such as PD [32, 37, 39], MS [35, 38], IBD [28], SLE [36], symptomatic knee osteoarthritis [31], fibromyalgia [34], and stroke survivors [30].

Therefore, in-depth analyses of the associations between real-world gait and abnormal fatigue in NND and IMID are currently lacking. The current study will analyse free-living data from several differing disease cohorts and will conduct an extensive exploration of various measures of gait and walking activity as a preliminary assessment of data collected by IDEA-FAST [6].

This study aims to:

(i) comprehensively explore the feasibility of using macro and micro gait characteristics from an IMU attached to the lower back to objectively identify PRO scores of physical and mental fatigue in NDD and IMID participants;
(ii) assess the associations of the macro and micro characteristics with selected PROs using a generalised linear mixed effect model and low vs. high fatigue binary classification performances of popular machine learning models;
(iii) explore the usefulness of the gait-model’s physiological feature groups using the associations from the linear mixed effect model.

## Materials and methods

### Experimental protocol

A total of 131 participants wore devices measuring activity for the IDEA-FAST feasibility study. All participants provided written informed consent and research was conducted in accordance with the Declaration of Helsinki and was approved by the London Riverside Ethics Committee (20/PR/0185) and the Health Research Authority (HRA). The inclusion criteria ensured the participants were over 18 and were willing and able to participate. The exclusion criteria ensured participants had no diagnoses or symptoms relating to their disorder that could interfere with the aims of the study (e.g., sleep disorder, chronic fatigue, etc.) [40]. The current study analysed data from a lower-back IMU approximately at the level of the L5 vertebra for two periods of five consecutive days in a free-living environment. The IMU was the McRoberts [41] Dynaport device, from which only the triaxial accelerometer data was used, with a sampling rate of 100 Hz and a range of ± 8 g (1 g is equivalent to 9.81 m/s^2^). Whilst wearing the device, participants were asked to complete short PROs up to four times a day: morning (09:00–12:00), early afternoon (13:00–16:00), late afternoon (17:00–20:00), and evening (21:00–24:00) on a smart phone provided. This included two questions asking how the participants were feeling with regards to their physical and mental fatigue (Likert items on a scale of 0–6). These data were collected between August 2020 and August 2021, therefore during the COVID-19 pandemic. More information can be found in [42] and a table of the participants’ demographics can be found in Table 1 in the Results section.

### Data processing

To assess the gait characteristics from these accelerometer data, the walking bouts, defined as periods of walking with three or more steps taken [43–46], were identified using algorithms validated with healthy adults [47]. From which, macro and micro measures of gait [14, 33, 48] were extracted using algorithms (including the walking bout detection algorthm used in the current analysis) that were validated with healthy older adults [49], PD and healthy controls in the real-world [50], ataxia and healthy controls [51], and post-stroke [52]. Statistical characteristics that represent these gait measures across the selected time-period were compared to the PROs to explore their relationships. The spread and balance of the PRO scores themselves were also analysed. Since the individual cohort sizes were small—as few as 9 participants—the analysis described below was performed on all disease cohorts pooled together, allowing for the exploration of a mixed-disease biomarker. Additionally, the outcomes of analyses with 30 healthy controls included in the data can be found in the Supplementary Materials.

#### Identification of bouts of walking

The raw accelerometer data were visually inspected to observe the orientation of the sensor and identify any periods of missing data. The sensor’s x–y-z axes from the raw accelerometer data were then renamed to give the participants vertical (VV), medial–lateral (ML), and anterior–posterior (AP) orientations. To further ensure the device was attached in the correct orientation, the mean of the sensor’s x-axis (participants’ vertical axis) was checked: if the mean across a day was positive, the data were visually inspected, and if required, the orientation was corrected. The participant was considered as more likely to be walking if moving on all three axes and the trunk was upright. To find the walking bouts, the accelerometer data were mean normalised, filtered with a second order lowpass digital Butterworth filter with a frequency cutoff of 17 Hz, and segmented into 0.1 s non-overlapping windows. Windows that met the conditions in Eqs. 1 and 2 were considered to contain data where the participant was upright and moving, where ${\sigma }_{AccVV}$ is the standard deviation (SD) of the accelerometer’s VV axis, ${\sigma }_{AccML}$ is the SD of the ML axis, ${\sigma }_{AccAP}$ is the SD of the AP axis, and $\overline{{Acc }_{VV}}$ is the mean of the accelerometer’s VV axis [47].

1 $${\sigma }_{AccVV}+{\sigma }_{AccML}+{\sigma }_{AccAP}\ge 0.05$$

2 $$\overline{{Acc }_{VV}}\le -0.77$$

The thresholds used in Eqs. 1 and 2 were defined in the validation study for this algorithm [47]. The neighbouring windows containing walking data were merged to give the bouts. Bouts that were very short and very close together (less than 20 windows: 2 s) were merged and bouts that lasted less than 20 windows were excluded [47]. The resulting walking bouts that began up to 2 h before the participant completed a PRO were selected for analysis. The macro and micro gait characteristics were then extracted from the original accelerometer data using these defined walking bouts.

#### Detection of steps

With the walking bouts identified, the steps were identified from the lower-back accelerometer data by finding the initial contact (IC) and final contact (FC) using MATLAB®, with the method outlined in [53]. The accelerometer data in the participant’s VV axis was detrended using the detrend function, mean normalised and filtered with a fourth order lowpass digital Butterworth filter with a frequency cutoff of 20 Hz. This uni-axial signal was integrated with cumtrapz and then differentiated using a Gaussian continuous wavelet transformation (CWT), thus smoothing the signal. IC events were then defined as the local minima of the CWT, detected using findpeaks, and FC events were defined as the local maxima from a further CWT differentiation [53]. Only IC events above the threshold of 0.4 $\times$ mean of the peaks and FC events above the threshold of 0.25 $\times$ mean of the peaks were considered. Detected walking bouts containing fewer than three steps were removed and gait initiation (first three and last five steps) was excluded from further analysis. To account for any outlier steps detected by the algorithm described, any steps associated with a step time ≤ 0.25s or ≥ 1.25s or a step length ≤ 0.23m or ≥ 0.95m, or any strides associated with a swing time ≤ 0.23s or ≥ 0.95s were removed from further analysis.

#### Macro gait characteristics

Macro characteristics are the measures of gait that reflect activity. Based on the gait model [14, 54], the gait characteristic groups for the walking (walking bouts) and non-walking (periods when walking was not detected) measures extracted from the accelerometer data were:

- *Walking volume*: number of steps in the walking bouts, length of the walking bouts (in seconds, excl. mean), total number of walking bouts, and number of walking bouts lasting 10 min or more;
- *Non-walking volume*: length of the non-walking bouts (in seconds, excl. mean) and number of non-walking bouts lasting 20, 30, and 50 min or more;
- *Pattern*: bout length pattern, mean walking time (s), and mean non-walking time (s);
- *Vector magnitude*: vector magnitude of the triaxial accelerometer (m/s^2^);
- *Variability*: variability of the bout lengths (s).

The variability of the bout lengths (${S}_{2}$) was calculated using the maximum likelihood technique (MLT) [55] because the accelerometer data were log normally distributed [56] and were found for both the walking (${S}_{2W}$) and non-walking (${S}_{2S}$) bouts [57].

The bout length pattern was calculated using the alpha parameter, which describes the distribution of the lengths of the walking bouts [57]. It was devised by Chastin et al. [58] and is derived using the MLT. The alpha parameter can be calculated with Eq. 3, for an array of bout lengths ${x}_{i}$ of length $n$.

3 $$\alpha =1+n {\left[\sum_{i=1}^{n}{\text{ln}}\frac{{x}_{i}}{{x}_{min}}\right]}^{-1}$$

The vector magnitude is the absolute magnitude of the acceleration of the device and was found using Eq. 4.

4 $$\text{vector magnitude}=\sqrt{{{Acc}_{VV}}^{2}+{{Acc}_{ML}}^{2}+{{Acc}_{AP}}^{2}}$$

The walking and non-walking bouts, steps taken during the walking bouts, and variability of the bout lengths were identified using MATLAB®, which were then exported, and the remaining measures were extracted with Python.

#### Micro gait characteristics

Micro characteristics are the measures of gait that reflect discrete gait outcomes. For these features, the gait model [14, 54] feature groups and the number of features within each group were:

- *Pace*: step velocity (m/s), step length (m), and SD of swing time (s);
- *Rhythm*: stance time (s), swing time (s), step time (s), and stride time (s);
- *Variability*: SD of the step time (s), SD of the stance time (s), SD of the step velocity (m/s), SD of the step length (m), and SD of the stride time (s);
- *Asymmetry*: asymmetry of the step time (s), swing time (s), and stance time (s);
- *Postural control*: asymmetry of the step length (m).

From the sequence of ${\text{IC}}_{i}$ and ${\text{FC}}_{i}$ events, the stance (duration the foot was in contact with the ground), stride (the full gait cycle, i.e., two successive steps), and swing times (duration the foot was not in contact with the ground) were found using Eqs. 5–7 [49].

5 $$\text{Stance time (s)}={\text{FC}}_{i+1}-{\text{IC}}_{i}$$

6 $$\text{Stride time (s)}={\text{IC}}_{i+2}-{\text{IC}}_{i}$$

7 $$\text{Swing time (s)}=\text{Stride time}-\text{Stance time}$$

The step length was found using Eq. 8 which is based in the inverted pendulum model [59]. Here, $h$ is the change in height of the participant’s centre of mass and $l$ is the pendulum length i.e., the sensor’s height above the ground, which was taken as 53% of the participant’s height [60].

8 $$\text{Step length (m)}=2\sqrt{2lh-{h}^{2}}$$

The change in height $h$ was found by a double CWT integration of the accelerometer’s VV axis. These position data were filtered with a fourth order highpass digital Butterworth filter with a frequency cutoff of 0.1 Hz to prevent integration drift [59].

With the step length, the step velocity can then be calculated using Eq. 9 [49].

9 $$\text{Step velocity (m/s)}=\frac{\text{Step length}}{\text{Step time}}$$

With these five gait characteristics extracted, the variation and asymmetry of each measure were also assessed. The variabilities were calculated by finding the SD of each gait characteristic, as seen in Eq. 10, and the asymmetries were calculated by finding the absolute difference between the left and right feet, as seen in Eq. 11 [49].

10 $${\text{Variability}}={\text{SD}}(\text{Gait characteristic})$$

11 $${\text{Asymmetry}}= \left|{\text{Gait characteristic}}_{\text{Left}}-{\text{Gait characteristic}}_{\text{Right}}\right|$$

For the asymmetry, the gait characteristics were separated into the left and right feet by automatically assigning the first detected step of each bout as right steps and alternating with the left step.

These micro characteristics were found using MATLAB and then exported to Python which was then used for the remainder of the analysis.

#### Gait measures

With the gait measures calculated, the features with multiple values within each 2-h period were pooled. Ten statistical characteristics were used to represent these metrics across the bouts that began up to two hours prior to the corresponding PRO. These were the mean, SD, variance, sum of the values, minimum and maximum value, median, and 25th and 75th percentiles. Measures, such as number of bouts, that only had one value across the 2-h period are represented in the results as ‘single’. This gave a total of 45 macro features and 58 micro features from the accelerometer for 1387 physical fatigue and 1380 mental fatigue PRO outcomes. Samples that did not have a PRO label nor any walking bouts associated were excluded. Therefore, participants whose entire data were excluded based on these criteria were also excluded from the analysis, giving 72 participants, plus an additional 30 healthy volunteers who have been included in analyses in the supplementary materials.

### Analysis of relationships between gait and abnormal fatigue

The relationships between the PRO scores and the digital measures gait described above were assessed using a generalised linear mixed effect model (GLMM) and machine learning classifiers. This was done by comparing each PRO score to the digital gait measures extracted from the two-hour period prior. This time period was selected since two-hours was deemed sufficient to capture walking behaviours and prior exploratory analysis indicated that two hours was a suitable length of time.

#### Generalised linear mixed effect model

Since the PRO scores were on a Likert scale and are therefore ordinal, the associations between the PROs and the gait measures described were assessed using generalised linear mixed effect model (GLMM). This analysis was implemented using the glmer function in R [61], which incorporates both fixed and random effect parameters in a linear prediction model using Eq. 12 [62].

12 $$g(E\left(Y|X,\alpha \right))= \eta +\alpha =X\beta +\alpha$$

Here, $X$ is the matrix of gait measures, $Y$ is the targets (i.e., the PRO scores), $\alpha$ is the random intercept, $E\left(Y|X,\alpha \right)$ is the estimate of $Y$ given $X$ and $\alpha$, $g(\cdot )$ is the link function for the mean of the distribution, $\eta$ is the mean of the distribution. Parameters $\beta$ and $\alpha$ were estimated using maximum likelihood estimation [62].

For the current analysis, the random effect was defined as the subject to account for repeated measures from each participant, the link function $g(\cdot )$ was binomial distribution, and the likelihood of a mixed effect model was approximated using adaptive Gauss-Hermite quadrature [61]. The samples were also weighted by the inverse of the number of samples with the same PRO score to prevent underprediction of the underrepresented scores.

The goodness-of-fit of the GLMM, and therefore the associations between each digital measure and their corresponding fatigue PROs, was evaluated with the model’s marginal and conditional R2 (as recommended by [63]), which theoretically range from 0 to 1, with 1 meaning a perfect fit. Marginal R2 is concerned with variance explained by the fixed factors, whereas conditional R2 is concerned by both the fixed and random factors [63].

#### Machine learning classifiers

To classify low vs. high fatigue using the macro and micro gait characteristics, two methods of splitting the data were used: intersubject cross validation (CV) (five-fold leave-subjects-out CV where 20% of the subjects were excluded as the test data) and intrasubject CV (five-fold cross validation within the data of each individual subject). For the intersubject method, the PROs were binarised by setting 0–2 as low fatigue and 3–6 as high fatigue [64]. For the intrasubject method, the PROs were binarised to low or high after splitting the data into the training/testing sets (five-fold CV) by setting threshold for binarisation as the mean of the training data. Therefore, the threshold for PRO binarisation was more reflective of the participants’ baseline, however one or two PROs from three participants had to be excluded since there was not enough range in the PRO scores to create two classes. These two methods allowed analysis of the macro and micro characteristics’ abilities to identify low/high rated PROs with an ‘unseen’ subject and for each individual subject.

For both methods, any missing features (macros: 6.5% of variability of the bout lengths; and micros: 0.5% of the step length and step velocity and 10.8% of the step length asymmetry) were replaced with the median of the training data. The median was selected since it less susceptible to extreme values than statistical measures such as the mean. Since the classes, in most cases, were imbalanced, the minority class was oversampled using a synthetic minority over-sampling technique (SMOTE) [65] from Imbalanced-learn [66]. The data were then scaled using StandardScaler [67] and shuffled. Once the data had been prepared, it was evaluated using four machine learning classifiers: support vector machine (SVM), k-nearest neighbours (kNN), random forest (RF), and Naïve Bayes classifier (NB). These models were selected since they represent a range of basic ideas in machine learning (separation, clustering, decision trees, and probability) and were implemented using the scikit-learn python package [67].

SVMs were introduced in 1995 [68] and classify by searching for an optimal hyperplane that separates the classes. If the data are separable, the hyperplane maximises a margin around itself that does not contain any data, creating boundaries for the classes. Otherwise, the algorithm establishes a penalty on the length of the margin for every observation that is on the wrong side. The SVM classifiers used in this analysis used an RBF kernel, which maps the data onto a higher dimensional space, where linear separation is then performed. The RBF kernel $K$ between two patterns $x$ and $x{\prime}$ is calculated using Eq. 13, where $\gamma$ is the hyperparameter defined by grid search as described below.

13 $$K\left(x,x^{\prime}\right)=exp\left(-\gamma {\Vert x-x^{\prime}\Vert }^{2}\right)$$

The kNN algorithm is based on the idea that similar groups will cluster. Training can be considered as ‘plotting’ training observations in a multidimensional feature space. The algorithm works by ‘plotting’ the testing observations and classifying them based on the class(es) of the nearest neighbour(s) within that feature space.

RF was introduced by Breiman [69] and is based on randomised decision trees. Decision trees are flowchart-like structures that predict the value of a target variable by learning a series of simple decision rules based on the training data. RF uses an ensemble of trees, each with a different random subset of the training samples and features. This decreases the variance, compared to an individual decision tree, and reduces the risk of overfitting. The class was then taken as the average of the trees’ probabilistic predictions, whereas the original publication [69] let each tree vote for a single class.

NB implements the Gaussian Naïve Bayes algorithm, where the likelihood of the features is assumed to be Gaussian (normally) distributed. Given a class variable $y$ and a feature vector ${x}_{1},{x}_{2}\dots {x}_{n}$, the predicted class $\widehat{y}$ is found using Eq. 14 [67].

14 $$\widehat{y}=\text{arg }\underset{y}{{\text{max}}}P(y)\prod_{i=1}^{n}P\left({x}_{i}|y\right)$$

Maximum A Posteriori (MAP) estimation is used to estimate the probability of class $y$, $P(y)$, and $P\left({x}_{i}|y\right)$. For Gaussian NB, the likelihood of features ${x}_{i}$ given class $y$, $P\left({x}_{i}|y\right)$, is calculated using Eq. 15, where ${\sigma }_{y}$ and ${\mu }_{y}$ are estimated using maximum likelihood [67].

15 $$P\left({x}_{i}|y\right)= \frac{1}{\sqrt{2\pi {\sigma }_{y}^{2}}}{\text{exp}}\left(-\frac{{\left({x}_{i}-{\mu }_{y}\right)}^{2}}{2{\sigma }_{y}^{2}}\right)$$

The parameters for these machine learning models were defined using a grid search with the training data of each fold. Tenfold cross validation was used to find the parameter combinations that returned highest balanced accuracy, which then defined these parameters for the final model of the fold. The parameters defined for the SVM were regularisation parameter (values tested: 0.001, 0.01, 0.1, 1, 10, 100, 1000) and the kernel coefficient for the RBF, $\gamma$, (values tested: 0.001, 0.01, 0.1, 1, 10, 100, 1000). For the kNN, the number of nearest neighbours, $k$, tested were 1, 2, 3, 4, 5, 6, 8, 10, 15. For the RF, the parameters tested were number of estimators (trees in the forest) (values tested: 5, 10, 50, 100, 200, 500) and the number of features to consider when looking for the best split (values tested: $\sqrt{N}$, ${{\text{log}}}_{2}N$, and $N$ (where $N$ is the number of features)).

Precision, recall, and balanced accuracy were used to evaluate the classifiers’ predictions on test data. Since the data were imbalanced, these metrics were selected as they avoid inflated performance metrics on imbalanced datasets. Equations 16–18 show the calculations for these performance metrics [67].

16 $${\text{precision}}=\frac{\text{TP}}{{\text{TP}}+{\text{FP}}}$$

17 $${\text{recall}}= \frac{\text{TP}}{{\text{TP}}+{\text{FN}}}$$

18 $$\text{balanced accuracy}= \frac{1}{2}\left(\frac{\text{TP}}{{\text{TP}}+{\text{FN}}}+\frac{\text{TN}}{{\text{TN}}+{\text{FP}}}\right)$$

where ${\text{TP}}$ is the true positive rate, ${\text{TN}}$ is the true negative rate, ${\text{FP}}$ is the false positive rate, and ${\text{FN}}$ is the false negative rate. Here, high fatigue is the positive class and low fatigue is the negative class for both the physical and mental fatigue PROs.

### Feature importance

To further investigate the impacts that these features have on the machine learning classifiers and to provide some clarity into the black-box nature of these models, the importance of the micro and macro feature characteristic groups was evaluated. This was done with a light gradient boosting machine (LGBM)—a decision tree-based model—and permutation feature importance.

#### Light gradient boosting machine

LGBM was introduced in 2017 [70] as an improvement on a typical gradient boosting decision tree: an ensemble model where the trees are in a series and each new tree minimises the errors of the previous tree [70]. The novel improvements of the LGBM are gradient-based one-side sampling (GOSS) and exclusive feature bundling (EFB). GOSS keeps all the instances with large gradients (errors) and performs random sampling on the instances with small gradients, since these instances are already well trained. EFB reduces the number of features by bundling mutually exclusive features into a single feature. Thereby, considerably speeding up the training of the model without compromising the accuracy.

The LGBM firstly splits the features into discrete bins to construct feature histograms during training. The feature importance is then calculated finding the Gini index of each node (decision rule). The Gini index of a node is calculated using Eq. 19, where ${n}_{j}$ is the importance of node $j$, ${w}_{j}$ is the weighted number of samples reaching node $j$, ${C}_{j}$ is the impurity value of node $j$ [71], $A(j)$ denotes the child node from class $A$ split on node $j$, and $B(j)$ denotes the child node from class $B$ split on node $j$ [72].

19 $${n}_{j}={w}_{j}{C}_{j}-{w}_{A\left(j\right)}{C}_{A\left(j\right)}-{w}_{B(j)}{C}_{B(j)}$$

Equation 20 can then be used to find the feature importance. Here, ${f}_{i}$ is the importance of feature $i$.

20 $${f}_{i}=\frac{{\sum }_{j:{\text{node}} j \text{splits on feature} i}{n}_{j}}{{\sum }_{k\in \text{all nodes}}{n}_{k}}$$

The average importance across the decision trees and cross validation folds were taken to give the importance of each feature. This LGBM ranker was implemented using the lightgbm package [71].

#### Permutation importance

Permutation feature importance was also used to analyse the macro and micro characteristics and was implemented using scikit-learn [67]. A model $m$ was fitted using the data $D$, and then a reference score $s$ was calculated. Here, the balanced accuracy was used as the score $s$. Each feature $j$ to be assessed was then permutated (randomly shuffled) to corrupt the samples and give the corrupted data ${\widetilde{D}}_{k,j}$. The balanced accuracy ${s}_{k,j}$ of model $m$ with this corrupted data was then computed. This process of permutating and calculating score ${s}_{k,j}$ was repeated $K$ times with iteration $k$. The importance ${i}_{j}$ of feature $j$ is then defined using Eq. 21 [73].

21 $${i}_{j}=s-\frac{1}{K}\sum_{k=1}^{K}{s}_{k,j}$$

As such, a higher value of ${i}_{j}$ indicates to a higher importance for that feature and negative values indicate that the model improved with the feature’s corruption, therefore it has negative importance. For this analysis, $K=$ 5 and the macro and micro characteristics data were analysed separately. The mean importance across the four classifiers described above was taken as the final permutation importance.

## Results

### Clinical and demographic characteristics

The data used in this analysis were collected at four clinical sites in Europe as part of the IDEA-FAST feasibility study [40]. The participants included healthy controls (HC = 30) and those with six different NDD and IMID conditions: Parkinson's disease (PD = 25), Huntington's disease (HD = 14), rheumatoid arthritis (RA = 24), systemic lupus erythematosus (SLE = 18), primary Sjogren’s syndrome (PSS = 18), and inflammatory bowel disease (IBD = 18).

The demographics of the participants who had usable data can be found in Table 1 and the number of samples from each cohort at each time of day is shown in Table 2. The disease cohorts (DC) data were used in the following analysis. The outcomes including the HC can be found in the supplementary materials.

**Table 1:** Demographics of the participants used in this study

Demographic = Population	Age (Years)	Sex (M/F)	Height (m)	Weight (kg)	BMI (kg/m^2^)
PD = 13	60.08 ± 11.34	7/6	1.75 ± 0.10	75.05 ± 12.56	24.40 ± 2.38
HD = 9	45.67 ± 11.17	4/5	1.74 ± 0.08	81.78 ± 18.73	27.47 ± 8.35
RA = 12	60.08 ± 10.43	2/10	1.65 ± 0.14	81.37 ± 16.41	30.45 ± 8.11
SLE = 9	51.33 ± 15.14	0/9	1.64 ± 0.07	69.36 ± 12.79	26.06 ± 5.38
PSS = 14	62.29 ± 12.62	2/12	1.62 ± 0.09	68.22 ± 8.23	26.27 ± 4.33
IBD = 15	35.60 ± 11.41	7/8	1.75 ± 0.11	74.79 ± 14.03	24.49 ± 3.50
DC = 72	52.51 ± 15.45	22/50	1.69 ± 0.12	74.84 ± 14.09	26.34 ± 5.61
HC = 30	48.70 ± 15.35	15/15	1.75 ± 0.10	79.72 ± 15.69	25.87 ± 4.24

Reported values are mean ± SD, aside from Sex which are the male to female ratio. *DC* Disease cohorts; *M* Male, *F* Female, *BMI* Body mass index

**Table 2:** Number of samples used in the current analysis

Demographic = Population	Morning		Early Afternoon		Late Afternoon		Evening	
	PF	MF	PF	MF	PF	MF	PF	MF
PD = 13	39	39	35	34	37	33	30	29
HD = 9	30	27	28	27	25	25	21	21
RA = 12	58	57	60	58	61	56	51	50
SLE = 9	58	57	50	53	49	51	56	59
PSS = 14	85	81	92	87	103	94	82	79
IBD = 15	69	76	84	92	88	96	96	99
DC = 72	339	337	349	351	363	355	336	337
HC = 30	100	87	107	103	121	120	125	110

*DC* Disease cohorts, *PF* Physical fatigue, *MF* Mental fatigue; morning: 09:00–12:00, early afternoon: 13:00–16:00, late afternoon: 17:00–20:00, evening: 21:00–24:00

Table 2 shows that there is not a large disparity between the number of samples for the two types of fatigue and four times of day, therefore these are not aspects that must be considered with high criticism throughout this analysis. There is, however, a substantial disparity between the number of samples from each disease cohort. Therefore, the findings from this study may be more applicable to those with PSS or IBD than those with SLE or HD.

#### PRO scores

**Fig. 1:**
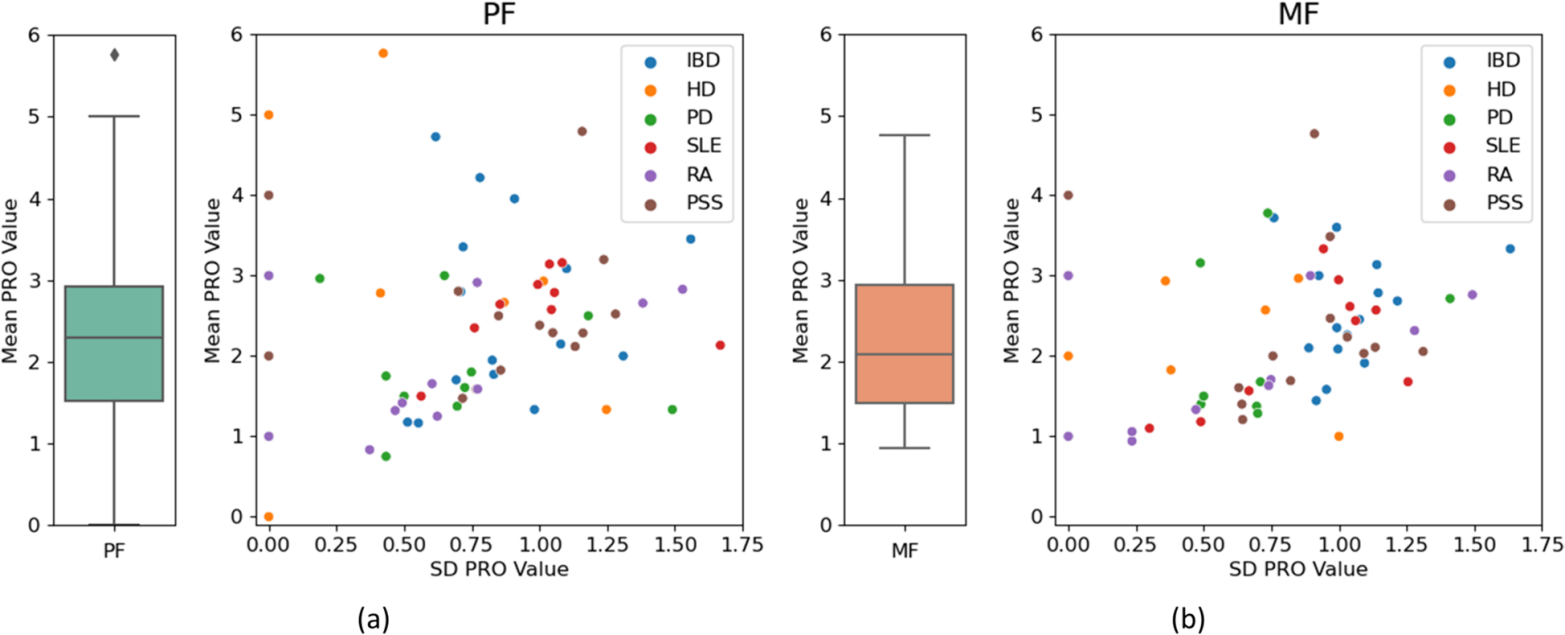
Summaries of the PRO scores (excluding healthy). **a** shows physical fatigue and **b** shows mental fatigue. Left: Box plots of the participants’ mean PRO scores. Right: Scatter plots of the mean against the SD for each participant’s PRO scores, colour represents cohort. Box plots show the median, interquartile range, 1.5 $\times$ interquartile range, and outliers of the data. *PRO* Patient reported outcome, *PF* Physical fatigue, *MF* Mental fatigue, *SD* Standard deviation

Figure 1 shows the physical and mental scores of the participants. The participants’ mean PF score shows more spread across the full breadth of the 0–6 scale. Whereas for MF, the scores are only within 1–5, therefore capping off the extremes. While the participants’ mean PRO scores have similar interquartile ranges for both fatigue types, the MF has a lower median closer to 2 whereas PF is closer to 2.5. The scatter plots of the PROs’ means against SDs also show that PF has more spread than MF. Furthermore, both fatigue types show a slight positive relationship; generally, the higher the mean PRO score for a participant, the higher the SD. There is very little clustering of the patient cohorts; the MF shows that IBD generally has higher SDs and HD has lower SDs than the other cohorts, which is not seen with the PF. Furthermore, the cohorts with higher PF means (score > 3.5) are IBD, HD, and PSS, and the two highest MF means are from the PSS cohort.

**Fig. 2:**
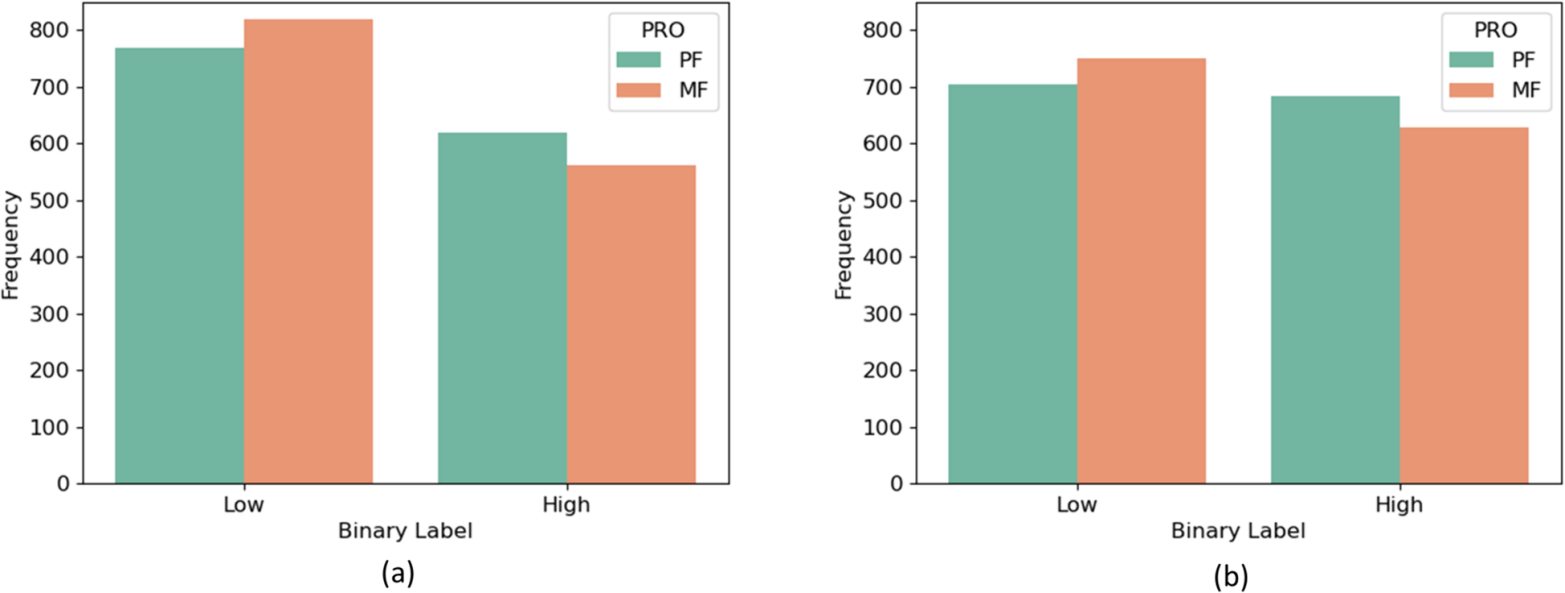
The number of low and high fatigue samples in each class (excluding healthy). **a** Binarisation threshold was two (intersubject method). **b** Binarisation threshold was the mean PRO value of each subject (intrasubject method). *PRO* Patient reported outcome, *PF* Physical fatigue, *MF* Mental fatigue

Figure 2 shows the number of samples in each class for both the inter- and intra-subject cross-validation methods. These plots show that defining the threshold as the mean of each subject gave more balanced classes than using a threshold score of 2. In both cases there are more MF than PF samples in the low class and fewer MF in the high class.

### Associations between micro and macro gait characteristics and PROs

A table of all associations between the gait characteristics and PROs from the GLMM and their corresponding p-values of the macro and micro characteristics can be found in the Supplementary Materials. Table 3 summarises the absolute associations to assess the relationships of the macro and micro characteristics with the two PROs. Overall, the associations are very weak. Fewer than an eighth of features had a statistically significant association with either PRO for both the macro and micro characteristics, all conditional R2 values were less than 0.2, and all marginal R2 values were less than 0.002. The variability of the non-walking bout lengths (in seconds) returned the strongest conditional R2 value with PF, 0.165, and the strongest marginal R2 value with MF, 0.0018.

**Table 3:** Summary of the linear mixed effect model associations and their statistical significance

Feature Group (No. of Features)	% of Statistically Significant Features (p < 0.05)		Mean absolute association				Strongest association			
			Conditional R2		Marginal R2		Conditional R2		Marginal R2	
	PF (%)	MF (%)	PF	MF	PF	MF	PF	MF	PF	MF
Macros [45]	6.7	11	0.157	0.096	0.0002	0.0003	0.165	0.100	0.0014	0.0018
Micros [57]	0.0	0.0	0.157	0.096	0.0002	0.0002	0.162	0.098	0.0010	0.0006

*No.* Number, *PF* Physical fatigue, *MF* Mental fatigue

**Fig. 3:**
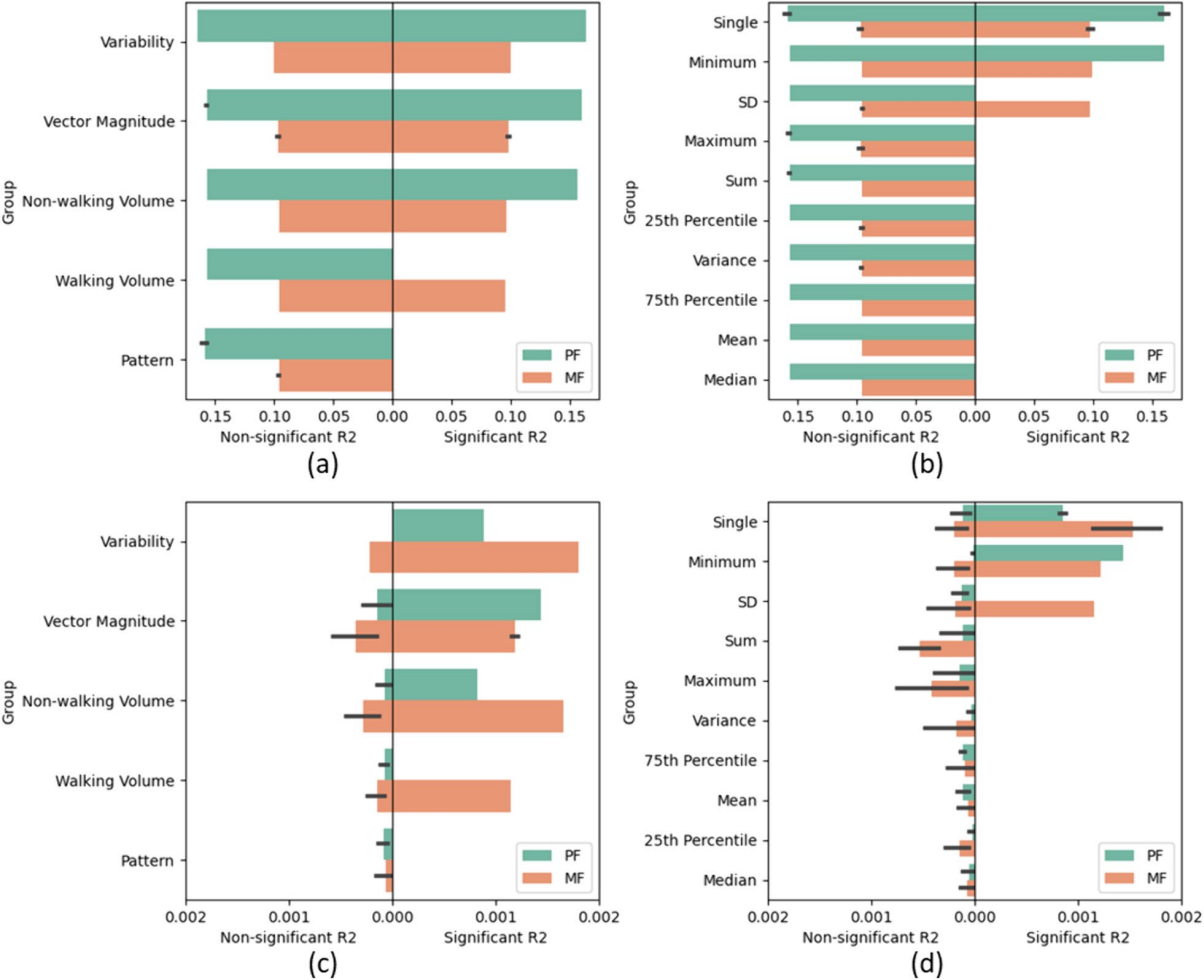
Bar plots of the R2 values from the GLMM of the macro characteristics with non-significant associations on the left and statistically significant associations on the right (p < 0.05). **a**, **b** show the conditional R2 and **c**, **d** show the marginal R2. **a**, **c** Gait characteristic group rank averaged across the statistical measures. **b**, **d** Statistic rank averaged across the gait characteristic groups. Features are ranked from highest to lowest mean R2 across the significant and insignificant measures, double weighted to the significant measures. Error bars show the 95% confidence interval. *PRO* Patient reported outcome, *PF* Physical fatigue, *MF* Mental fatigue, *SD* Standard deviation, *GLMM* Generalised linear mixed effects model

**Fig. 4:**
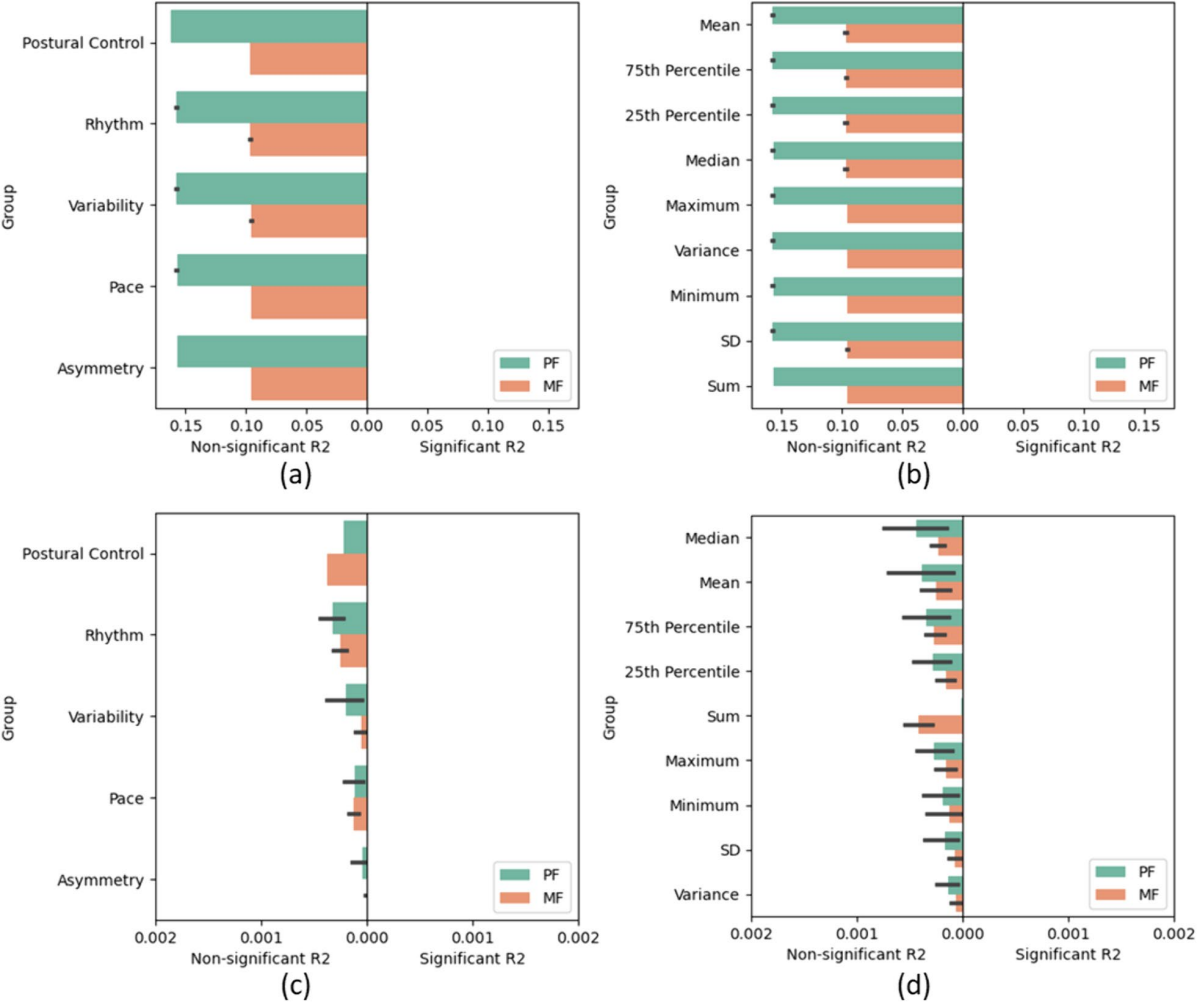
Bar plots of the R2 values from the GLMM of the micro characteristics with non-significant associations on the left and statistically significant associations on the right (p < 0.05). **a**, **b** show the conditional R2 and **c**, **d** show the marginal R2. **a**, **c** Gait characteristic group rank averaged across the statistical measures. **b**, **d** Statistic rank averaged across the gait characteristic groups. Features are ranked from highest to lowest mean R2 across the significant and insignificant measures, double weighted to the significant measures. Error bars show the 95% confidence interval. *PRO* Patient reported outcome, *PF* Physical fatigue, *MF* Mental fatigue, *SD* Standard deviation, *GLMM* Generalised linear mixed effects model

Figures 3 and 4 show the R2 values of the GLMM for each macro and micro characteristic. The measures were ranked based on the mean R2 values, with the statistically significant (p < 0.05) measures double-weighted since these associations are more meaningful. The statistics were also ranked to explore any influence these measures may have on the findings. When considering both figures, there are minute differences between conditional R2 values, whereas the marginal R2 values are more variable.

Figure 3 shows that, generally, the conditional R2 had stronger associations with the PF, whereas the marginal R2 had stronger associations with the mental fatigue. For both the conditional and marginal R2, all measures of gait pattern were not statistically significant (p ≥ 0.05). After which, the two groups with the weakest associations were the walking then non-walking volume, with the strongest associations from the bout length variability followed by the vector magnitude. The three highest ranked statistics were the single-value measures, followed by the minimum and SD, for both conditional and marginal R2.

Figure 4 shows that none of these gait measures were statistically significant (p ≥ 0.05). With the ranks based purely on the non-significant measures, both the conditional and marginal R2 agree on the order of the gait characteristics—postural control, rhythm, variability, pace, then asymmetry—and the best statistic was the mean. Additionally, the conditional R2 returned higher (but non-significant) R2 values with the PF than MF in every instance.

### Machine learning classifier performances

**Fig. 5:**
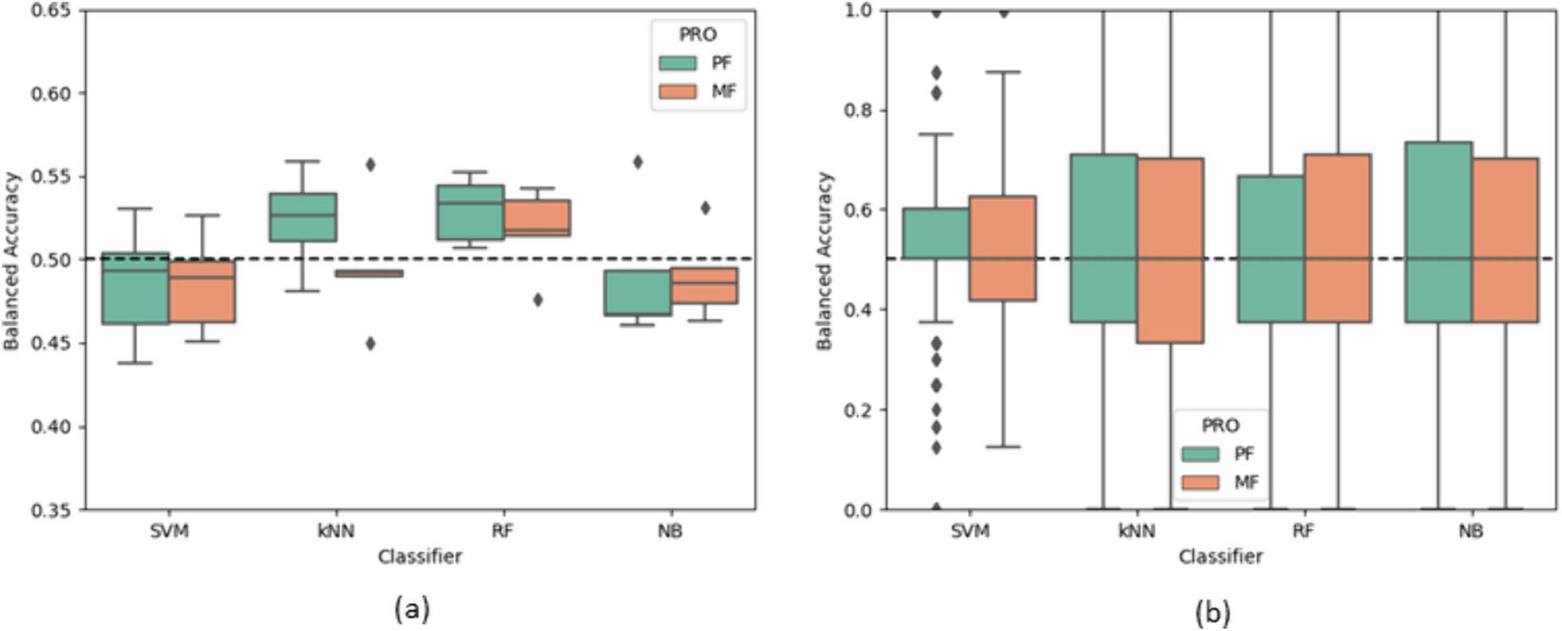
Boxplots of the balanced accuracies of the classifiers for all folds with the gait macro characteristics. **a** Cross validation across the subjects (Intersubject method). **b** Cross validation within each individual subject (Intrasubject method). The dashed line represents random chance (50%). *PRO* Patient reported outcome; *PF* Physical fatigue, *MF* Mental fatigue, *SVM* Support vector machine, *kNN* k-nearest neighbours, *RF* Random Forest, *NB* Naïve Bayesian

**Fig. 6:**
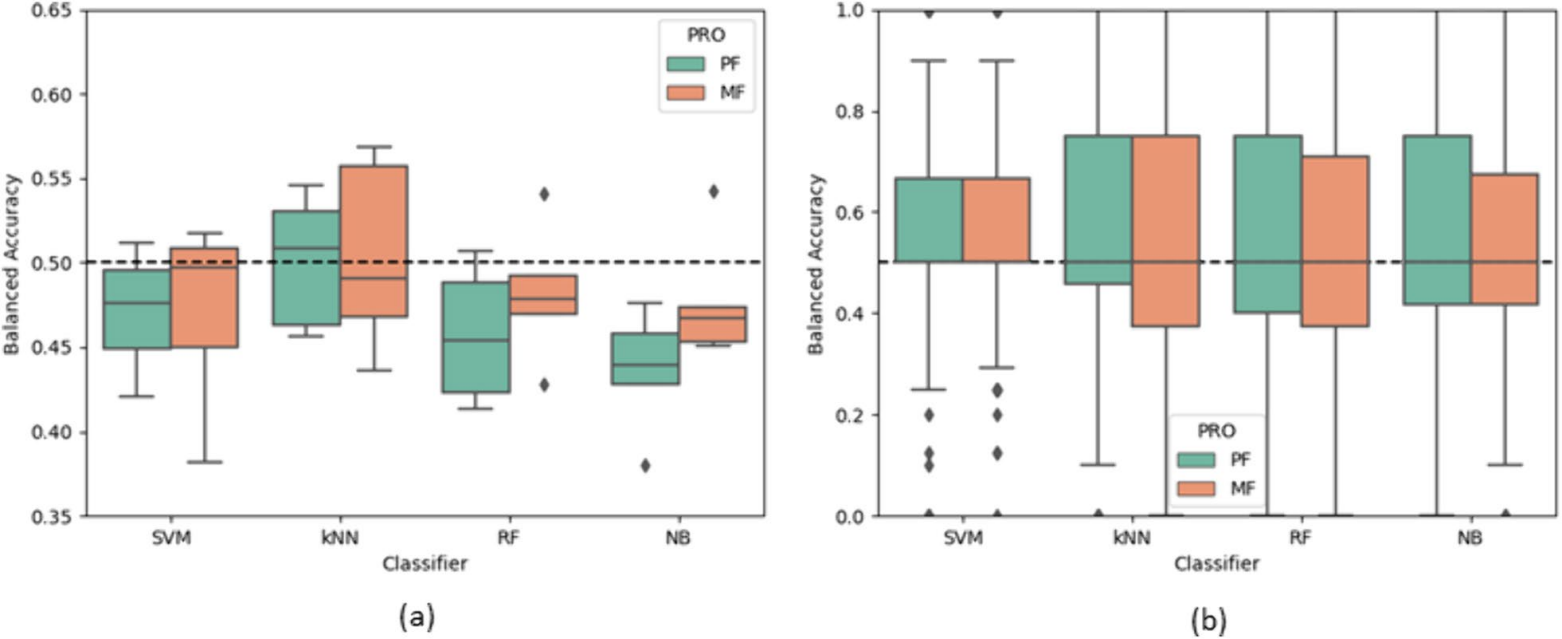
Boxplots of the balanced accuracies of the classifiers for all folds with the gait micro characteristics. **a** Cross validation across the subjects (Intersubject method). **b** Cross validation within each individual subject (Intrasubject method). The dashed line represents random chance (50%). *PRO* Patient reported outcome, *PF* Physical fatigue; *MF* Mental fatigue, *SVM* Support vector machine, *kNN* k-nearest neighbours, *RF* Random Forest, *NB* Naïve Bayesian

Figures 5 and 6 show the outcomes of the classifiers trained on the macro and micro characteristics, respectively. Overall, the classification accuracies are around 50% (random chance) for both feature groups and CV methods. The kNN and RF outperformed the SVM and NB for the intersubject method with the macro characteristics, and the micro characteristics generally returned lower accuracies than macro for this CV method. Whereas, the micro characteristics’ interquartile ranges are generally higher for the intrasubject CV method, although all medians were 50% for both feature groups for this CV method. The interquartile range and whiskers for the intrasubject CV method is much larger than the intersubject CV, possibly due to there being over 100 folds for intrasubject CV, compared to five.

**Fig. 7:**
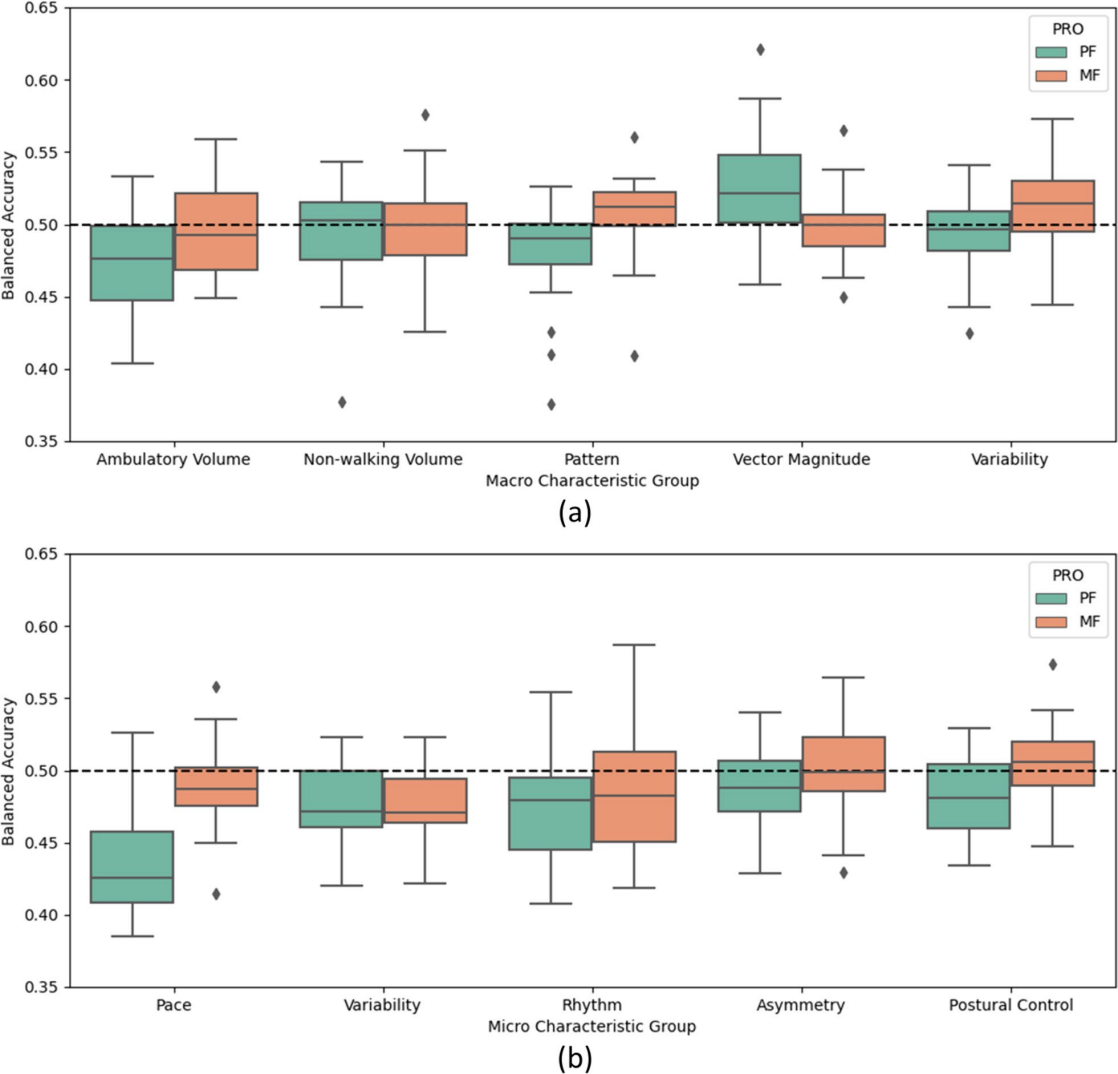
Boxplots of the balanced accuracies of the classifiers for all folds with each individual gait micro characteristic group and the intersubject method, across each fold and classifier. The dashed line represents random chance (50%). **a** Macro gait characteristics. **b** Micro gait characteristics. *PRO* Patient reported outcome, *PF* Physical fatigue, *MF* Mental fatigue

Figure 7 shows the performance of the classifiers when trained on the characteristic groups separately for the macro (a) and micro (b) features. These plots also show that the balanced accuracies were around chance (50%), without a substantial difference between the characteristic groups for both the macros and micros. In most cases the MF returned slightly higher or similar accuracies, with the vector magnitude as the exception, where the accuracies for physical fatigue are as high as 62.08. The two lowest performing groups were walking volume and pace, especially for the physical fatigue.

Table 4 shows a summary of the performances of the classifiers by reporting the mean balanced accuracies across the fold for each model. The precisions and recalls of the analyses in this section can be found in the supplementary materials. Overall, the accuracies were similar to chance. When considering the intersubject CV method, vector magnitude with RF returned the highest performance for PF with 54.47% (precision = 48.2%, recall = 48.6%), and for MF the highest accuracy was with postural control with RF with 51.89% (precision = 42.6%, recall = 44.9%). For the intrasubject CV method, the micros with the SVM classifier returned the highest classification accuracy in both cases with 56.90% (precision = 43.9%, recall = 51.4%) and 55.74% (precision = 42.7%, recall = 49.3%) for PF and MF, respectively.

**Table 4:** Summary of the balanced accuracies of the machine learning classifiers

Train-Test Split	PRO	Group	Mean Accuracy		Highest Accuracy	
			PF	MF	PF	MF
Intersubject CV method	Macros	All	50.69 ± 2%	49.70 ± 1%	52.98%	51.69%
		Walking Volume	47.35 ± 1%	49.56 ± 0%	48.85%	49.95%
		Non-walking Volume	49.12 ± 3%	49.62 ± 1%	50.71%	51.39%
		Pattern	47.91 ± 1%	50.64 ± 1%	49.73%	51.72%
		Vector Magnitude	**52.73** ± 1%	49.99 ± 1%	**54.47%**	50.60%
		Variability	49.29 ± 1%	**51.10** ± 1%	50.02%	**51.86%**
	Micros	All	46.62 ± 3%	48.36 ± 1%	50.09%	50.42%
		Pace	43.62 ± 2%	49.11 ± 2%	45.53%	51.39%
		Variability	47.40 ± 2%	47.37 ± 2%	49.85%	49.02%
		Rhythm	47.58 ± 2%	48.50 ± 2%	49.83%	50.86%
		Asymmetry	**48.85** ± 1%	49.82 ± 1%	49.84%	51.81%
		Postural Control	48.09 ± 2%	**50.55** ± 1%	**50.72%**	**51.89%**
Intrasubject CV method	Macros	All	53.44 ± 1%	53.21 ± 1%	54.54%	54.53%
	Micros	All	**56.13** ± 0%	**54.70** ± 1%	**56.90%**	**55.74%**

Reports the mean ± SD of the balanced accuracies averaged across each fold and the maximal mean balanced accuracy given by a classifier. Bold denotes the 'best’ outcome for each PRO, feature group, and cross-validation approach. *PRO* Patient reported outcome, *PF* Physical fatigue, *MF* Mental fatigue

Generally, the intrasubject method returned higher accuracies than intersubject and for the macro and micro characteristics each combined in ‘All’, the macros outperformed the micros, especially for PF. Overall, the differences in performances between the characteristic groups were considerably small, especially when considering the SDs.

### Feature importance

To further investigate the usefulness of the different inputs, the importance of the features were ranked using two machine learning-based rankers: LGBM tree ranker, and permutation importance. However, due to the poor performance of the machine learning classifiers, the outcomes of these rankers may be misleading and are therefore not reported in this manuscript but are available in the supplementary materials.

## Discussion

To our knowledge, this is the first study to comprehensively investigate macro and micro gait characteristics in a free-living environment and their relationships with self-reported abnormal fatigue in multiple NDD and IMID cohorts and healthy participants. We have investigated the association between these gait measures and the PROs using a GLMM and binary machine learning classification and inspected the usefulness of the gait domains using the GLMM. Overall, the current analysis has shown weak relationships between digital measures of real-world gait and abnormal fatigue.

Generally, the balanced classification accuracies were low and the GLMM showed weak associations; the highest balanced accuracy (averaged across the CV folds) was 56.90% and the strongest association was 0.165 (for a range of 0 to 1). Therefore, the current analysis has shown that these conventional summary gait metrics of walking behaviour are poorly related to the patients’ perception of their fatigue. The analysis described in the current study was additionally conducted on all available participants i.e., including the healthy controls. The outcomes of these additional analyses can be found in the supplementary materials. Notably, we found that these additional participants did not substantially change the findings, therefore they did not introduce substantial noise, nor clarify the relationships.

### Machine learning classifier performances

The machine learning classifiers returned poor classification performances (< 57%), meaning these algorithms were unable to reliably identify the participants’ reported physical or mental fatigue from the macro and micro characteristics. This indicates that these gait measures have a weak relationship with patient reported fatigue. Figures 3b and 4b show plots of the classifier outcomes with intrasubject CV and display a wide range in balanced accuracies: 0% to 100% across the folds. This may be due to the limited variation in some participants’ reported fatigue severity during the study period. In most cases, the classification accuracies were higher when using the intrasubject CV approach, compared to intersubject CV (with all features). This may reflect the subjective nature of the PROs and how individual patients score their own levels of fatigue. For example, if a participant has consistently experienced high fatigue for an extended duration and has therefore acclimatised to these symptoms, their walking behaviours may have a different relationship with their fatigue, compared to other participants. This may also be due to the pooling of six different disease cohorts in the intersubject CV method causing large intersubject variability in both the gait behaviours and the participants fatigue perception. Additionally, this is also possibly due to the flexibility in the assignment of the low and high classes in the intrasubject CV approach. As seen in Fig. 2, the classes are much more balanced when defined by each participant’s mean PRO, as opposed to a universal threshold. Generally, the kNN and RF were the best classifiers, but the differences in performances from each classifier were subtle. Overall, the machine learning classifiers were unable to reliably separate low and high physical and mental fatigue with these data.

### Feature comparisons

When considering the outcomes of the GLMM and classifier performances, it is unclear whether the macro or micro characteristic features are more useful. There is no clear group with stronger associations from the GLMM, though none of the micro characteristics were statistically significant (p < 0.05), and the macros generally outperformed the micros with the intersubject CV method, but the micros generally outperformed the macros with the intrasubject CV method. This indicates that when exploring fatigue scores between participants at the population level, macro characteristics of gait may be more useful, whereas micro characteristics of gait may be more useful when exploring fluctuations in fatigue for individual participants. Furthermore, it is possible a combination of select macro and micro characteristics could increase classifier performances.

Looking at the feature rankings from the GLMM, there is little consistency for the statistical characteristics summarising the measures across the 2-h windows, compared to the gait characteristics. However, the single value measures were ranked at the most important by both the conditional and marginal R2. These measures include the number of walking and non-walking bouts, alpha parameter (pattern) and bout length variability, and since the pattern, walking and non-walking volumes were the lowest ranked of the gait characteristics, this ranking was likely influenced by the bout length variability, which was ranked the highest of the gait characteristics.

According to the R2 values from the GLMM, the feature groups with the highest importances in the macro characteristics were vector magnitude and bout length variability. One study investigated light and moderate/vigorous activity levels from the vector magnitude of a hip-worn accelerometer in 123 SLE participants [36]. The authors found associations (p < 0.05) with moderate/vigorous activity with fatigue PROs but not light activity. Therefore, future research could investigate the vector magnitude during higher and lower activity (such as walking) levels. There are examples of analysis of the relationship between fatigue and the micro-characteristic gait variability for adults with NDD or IMID in the literature [34, 74], but the analysis of the variability of the walking bout lengths themselves are not as popular. Therefore, this macro bout length variability would be an interesting point of further investigation.

For the micro characteristic feature groups, the current analysis found the feature groups with the overall highest ranks (although this was based on statistically non-significant measures (p ≥ 0.05)) were postural control (which only included one measure: the asymmetry of the step length) and rhythm. One study found that temporal variation of postural balance was predicted collectively by pain and fatigue (30.7%) (p < 0.001) in 15 RA participants [75]. For rhythm, one study found an association of higher scores of physical fatigue with step time (rhythm) in healthy older adults [33]. Since the literature is congruent with the findings of the current study, the current analysis can conclude that these measures could be more useful for future investigations.

Intuitively, it is reasonable to expect that higher fatigue would be associated with less activity, e.g. fewer steps and less time spent walking. However, the current analysis has found this to not be the case. Walking volume (step count, number of walking bouts, and length of walking bouts) was ranked second-to-last and PF did not have any statically significant associations, for both conditional and marginal R2. Furthermore, the walking volume had some of the lowest classification accuracies. Two studies found no statistically significant relationship between walking volume measures and abnormal fatigue: daily step count over a 48-h recording period in 53 post-stroke participants reported no relationship with fatigue from FSS and visual analogue scale (VAS) [30]; and the number of walking periods (> 10 s) and duration of dynamic activities over a 24-h period showed no difference between 10 fatigued (Checklist Individual Strength-fatigue (CIS-fatigue) score of ≥ 35) and 10 non-fatigued IBD participants [28]. However, one study found that for 45 PD participants, those with fatigue (from MDS-UPDRS, 17% of participants) had a lower step count (steps/day from 3-day recording periods) [37]. Therefore, most of the literature is congruent with the findings in the current analysis, though it is possible that the disease cohort can have an impact on the relationship between measures of walking volume and fatigue. We can conclude that there is potentially no overall relationship between walking volume and the abnormal fatigue in these NDDs and IMIDs. This conclusion is in line with the experiences reported by many patients with persistent fatigue symptoms. Therefore, if there is a relationship between walking and abnormal fatigue, it is possibly in how they walk, not how much. This highlights the necessity for digital wearables to analyse the more subtle characteristics of walking behaviours over longer recording periods, since these ‘simpler’ measures of gait that could be assessed visually by a clinician were insufficient for reliably identifying fatigue severity in the current analysis and existing literature.

### Limitations

The main limitation of the current analysis, and any analysis exploring fatigue, is the subjective nature of the PROs. They are not an objective measure of fatigue, rather a measure of the participants’ perception of their own fatigue symptoms. Therefore, the “ground truth” used to train and test the classifiers is not necessarily objective fact. Thus, highlighting the need for alternative, objective measures of fatigue. Additionally, the limited variation in some individuals’ PRO scores used in this study may have also had an impact on the outcomes reported in the current analysis. This limited variation may have been exacerbated by the use of six-point Likert scales, which may have been insufficient to appropriately describe the participants’ perception of their fatigue.

Another main limitation of this analysis is that, despite having one of the largest participant populations in the literature, the data used in current analysis contains a relatively small number of participants, thus limiting the representation of intersubject variability. Moreover, the individual disease cohorts were very small, with some containing as few as 9 participants, and as such analysis of these individual cohorts was not performed. To counteract these small cohorts, the analysis was done on all participants pooled together, which allows for the exploration for a mixed-disease biomarker, but also obscures the impacts of these individual disease types on the gait behaviours and the participants’ perception of their fatigue. Additionally, the algorithms used to identify the walking bouts and extract the macro and micro characteristics were not validated on all disease cohorts used within the current study. However, they were validated on healthy adults [49–51] and participants with varying mobility impairments, including Parkinson’s disease [50], ataxia [51], and post-stroke [52].

Furthermore, these data were collected during COVID-19 pandemic between August 2020 and August 2021. This may have, for instance, limited the amount of walking data available and been unrepresentative of many participants’ lifestyles outside of a pandemic situation. Consistently, the walking bouts were very short, only 25% of walking bouts lasted over 30 s, therefore very little continuous walking was represented in these data. Finally, factors such as medication use, time of day, age, and sex should be explored in future analysis.

### Future work

The main novelty of this work is the comprehensive analysis and comparison of these features for free-living data with multiple NDD and IMID cohorts. This was done as a preliminary assessment before analysis of the IDEA-FAST clinical observation study (COS), which is currently ongoing and plans to include 2000 participants. As a result, multiple avenues of future investigations that could be considered with the COS—and similar data sets—include investigating the disease cohorts separately, considering confounding factors, and including other attributes that may aid the machine learning classifiers. Confounding factors such as participant weight, age, sex, and medication can impact macro and micro gait features and therefore providing this information could assist the classifiers in intersubject CV. For intrasubject CV, a possible input is the time of day, since this may have an impact on the participants’ perception of their symptoms i.e., fatigue during midday may be rated as more severe than during the evening, as well as their activity levels. For instance, one study found that, in PD, those with fatigue had a distinctly lower step count in the morning and afternoon hours, but not during the evening hours [37]. However, these authors also found no interaction between time of day and patient fatigue (p = 0.08). Future research should explore other measures of mobility in addition to gait characteristics such as walking speed, turning, non-linear metrics, and signal-based features, along with exploring different granularities, different time windows, and exploiting the continuous nature of these data, as well as data driven approaches with deep learning. In addition, the gyroscope data collected by the IMU would by an interesting addition to these analyses. Another important factor for future investigations to consider is potential approaches to better handle the subjective nature of the PRO scores.

## Conclusions

The current analysis investigated the associations of macro and micro gait characteristics with patient reported physical and mental fatigue in six NDD and IMID cohorts. Overall, the associations and classification accuracies were low—all R2 values < 0.17 and all mean accuracies < 60%—thus indicating to the complexity of identifying objective correlates of symptoms of fatigue from changes in gait. Most notably, the walking volume (step count and time spent walking) was one of the lowest performing domains of the macro characteristics, indicating that, counterintuitively, the participants did not change their amount of activity with regards to their fatigue severity. These “traditional” gait measures used in the current analysis were insufficient for identifying patient reported fatigue, therefore further investigation is required for a mixed-disease biomarker. More “sophisticated” measures derived from a digital wearable could be more interesting for future work and could include accelerometer vector magnitude during lower and higher activity intensity, bout length variability, and measures of postural control and gait rhythm, which were found to be more useful by the current analysis. However, comparisons of patient cohorts and with a larger participant population is important for future investigations.

### Supplementary Information

Supplementary Material 1.

## Data Availability

The data for this study are available from the corresponding author on reasonable request and subject to the approval of the IDEA-FAST consortium.

## Abbreviations

AP: Anterior–posterior
BMI: Body mass index
CoV: Coefficient of variation
CV: Cross-validation
CWT: Continuous wavelet transform
DC: Disease cohorts
EFB: Exclusive feature bundling
F: Female
FC: Final contact
FSS: Fatigue severity score
GLMM: Generalised linear mixed effect model
GOSS: Gradient-based one-side sampling
HC: Healthy controls
HD: Huntington’s disease
IBD: Inflammatory bowel disease
IC: Initial contact
IMID: Immune-mediated inflammatory diseases
IMU: Inertial measurement units
kNN: K-nearest neighbours
LGBM: Light gradient boosting machine
M: Male
MF: Mental fatigue
MFI: Multidimensional fatigue inventory
ML: Medial–lateral
MLT: Maximum likelihood technique
MS: Multiple Sclerosis
NB: Naïve Bayesian
NDD: Neurodegenerative disorders
PCA: Principle component analysis
PD: Parkinson’s disease
PF: Physical fatigue
PRO: Patient reported outcome
PSS: Primary Sjogren’s syndrome
RA: Rheumatoid arthritis
RF: Random forest
RMCorr: Repeated measures correlations
SD: Standard deviation
SLE: Systemic lupus erythematosus
SMOTE: Synthetic minority over-sampling technique
SVM: Support vector machine
VV: Vertical
6MWDT: Six-minute walking test
10MWT: Ten-meter walking test
